# Supplementary material for: Cometary impacts an unlikely pathway towards the origins of life on Earth
Source: Sci Rep. 2026 Oct 28;16:31688. doi: 10.1038/s41598-026-74214-y (PMC13716819; doi:10.1038/s41598-026-74214-y)
Supplement: Supplementary file 1 [file 41598_2026_74214_MOESM1_ESM.pdf]

Supplementary Text

Jupiter-family comets' rotational spin-barrier

The vulnerability of cometary nuclei to rotational instability is evidenced by the so-called 'spin-barrier' – a critical rotation period below which large (gravity-dominated) comets fragment due to centripetal forces[1,2]. While previous studies have, in detail, calculated this critical period for both spheres[3] and triaxial ellipsoids[4], we motivate here this critical period following approximately the heuristic derivation presented in[5].

Consider a spherical nucleus comprising two hemispheres of radius R, with rotation period $P_{\rm rot}$. Each hemisphere's centre of mass will have radial velocity

$$v_r = \frac{3\pi R}{4P_{\rm rot}}, \tag{1}$$

and will therefore experience an outwards centrifugal acceleration

$$a_c = \frac{3\pi^2 R}{2P_{\rm rot}^2}. \tag{2}$$

When this acceleration exceeds both the comet's self-gravity and tensile strength, the comet will break-up; comets will therefore break-up with rotation periods smaller than

$$P_{\rm crit} = R\left(\frac{\rho\pi^2}{\sigma_T(1+\kappa)}\right)^{1/2} \tag{3}$$

where $\kappa = (64/81)\cdot\pi G\rho^2R^2/\sigma_T$, ρ is the comet's bulk density and σ_T its tensile strength. This is in agreement with the more detailed calculation in[3] to order unity.

It is clear from Equation 3 that this critical rotation period has very different behaviour in the large and small radius limits. For large comets, self-gravity dominates such that $P_{\rm crit}$ is independent of both size and tensile strength,

$$P_{\rm crit}^{\rm grav} \simeq \left(\frac{3\pi}{\rho G}\right)^{1/2} \simeq 4.26\,\left(\frac{\rho}{0.6\,{\rm g\,cm^{-3}}}\right)^{-1/2}\ {\rm hr}. \tag{4}$$

In contrast, small comets are strength-dominated with a critical rotation period dependent on both radius and tensile strength,

$$P_{\rm crit}^{\rm str} \simeq \left(\frac{\rho\pi^2}{\sigma_T}\right)^{1/2} R. \tag{5}$$

This critical rotation period is compared against modern JFCs[1] and small asteroids[6] in Fig. 1. All comets in this sample are stable against rotational instability when assuming tensile strengths of the order $1-10$ Pa. This is consistent with the typical tensile strength of cometary nuclei, which lie in the range $\sim 1-100$ Pa[7,8].

We further note that the median rotation periods of comets is significantly larger than that of asteroids[9,10], consistent with the much weaker tensile strength of cometary nuclei. The simplest explanation for these data is that the observed rotation period distributions reflect the density dependence of $P_{\rm crit}$, and that the current-day JFC population is strongly sculpted by the effects of rotational instability[10].

The timescale of rotational spin-up

It is well established observationally, in the form of significant non-gravitational accelerations, that the orbits of comets are perturbed by reactive forces in response to outgassing[11,12]. A net torque is a natural by-product of non-uniform mass loss (e.g., due to an asymmetric spatial distribution of vents[10,13]), which will change the direction and magnitude of a comet's spin angular momentum. Given that it is difficult observationally to determine a comet's spin axis, rotation period is typically used as a readily accessible proxy for this evolution[13–15].

Rotational spin-up is investigated in detail in Jewitt[10], using a sample of published rotation periods and period changes for JFC nuclei[16]. Here, we motivate the size-dependence of the timescale for outgassing to spin-up cometary nuclei to the point of rotational instability, following simple (largely dimensional) arguments.

We consider a spherical nucleus, radius R, experiencing a reactive force $F_{\rm ng}$ due to anisotropic outgassing. Its change in angular velocity $\Omega = |\Omega|$ will be given by

$$I\dot{\Omega} = \tau_\Omega, \tag{6}$$

where I is the comet's moment of inertia and $\tau_\Omega = \boldsymbol{\tau} \cdot \boldsymbol{\Omega}/\Omega$ is the component of the net torque $\boldsymbol{\tau}$ along $\boldsymbol{\Omega}$.

Intuitively, the net torque should be related to the magnitude of the reactive force and size of the comet ($\tau \sim R \times F_{\rm ng}$) via some effective (dimensionless) moment arm, ξ [e.g.,[10,14,15]], accounting for the fact that not all outgassed material will torque the comet. In the extreme cases, $\xi = 1$ corresponds to perfectly anisotropic (i.e., collimated) outgassing from the surface of the comet, while $\xi = 0$ corresponds to perfectly isotropic outgassing (generating no torque on the comet). The component of the net torque along $\boldsymbol{\Omega}$ is thus,

$$\tau_\Omega = \xi R F_{\rm ng}. \tag{7}$$

Assuming spherical symmetry ($I = 2MR^2/5$), the comet's angular velocity will therefore evolve as

$$\dot{\Omega} = \frac{5\xi F_{\rm ng}}{2MR}, \tag{8}$$

where M is the comet's mass.

It is natural to expect [and supported by observation of both short-and long-period comets[10,17]] in the sublimative hypothesis of outgassing, that activity (and hence the magnitude of the net force acting on the comet) will be proportional to nucleus surface area and depend on distance from the Sun as r^{-2}, such that

$$F_{\rm ng} = \alpha_{\rm ng} M_0 \left(\frac{R}{R_0}\right)^{-2} \left(\frac{r}{r_0}\right)^{-2}, \tag{9}$$

where $\alpha_{\rm ng}$ is the typical measured non-gravitational acceleration of short-period comets, R_0 (M_0) a characteristic comet radius (mass), and $r_0 \simeq 2.8$ au the characteristic orbital radius[12].

We therefore find

$$\dot{\Omega} = \frac{5\xi \alpha_{\rm ng}}{2R_0} \left(\frac{R}{R_0}\right)^{-2} \left(\frac{r}{r_0}\right)^{-2}. \tag{10}$$

Small comets are particularly susceptible to torques driven by anisotropic outgassing, and will be rapidly spun-up to the point of rotational disruption.

We can finally calculate the number of orbits (N) required to spin-up a comet to the point of rotational instability by considering

$$\int_{\Omega_{\rm init}}^{\Omega_{\rm crit}} d\Omega = \frac{5\xi \alpha_{\rm ng} r_0^2}{2R_0} \left(\frac{R}{R_0}\right)^{2} \int_0^{2\pi N} r^{-2} \frac{r^2 df}{na^2\sqrt{1-e^2}}, \tag{11}$$

where (n, a, e, f) are the characteristic mean motion, semi-major axis, eccentricity and true anomaly of short-period comets. The corresponding timescale of rotational instability is therefore

$$\tau_{\rm ri} = \frac{2\pi N}{n} = \frac{4}{5}\left(\frac{aR_0\sqrt{1-e^2}}{\xi \alpha_{\rm ng} r_0^2}\right)(\Omega_{\rm crit} - \Omega_{\rm init})\left(\frac{R}{R_0}\right)^2. \tag{12}$$

To estimate this timescale for (Earth-crossing) short-period comets ($a \sim 4\,$au, $e \sim 0.75$, $q \sim 1\,$au), assuming an effective lever-arm $\xi \sim 0.006$[10,15], and a typical non-gravitational acceleration $\alpha_{\rm ng} \sim 10^{-9}$ au d^{-2}[15,18], we find

$$\tau_{\rm ri} \sim 100 \left(\frac{R}{1\ \rm km}\right)^2 \ {\rm yr}, \tag{13}$$

recovering (to order of magnitude) the timescale found by Jewitt[10] – consistent with the observed spin-up of short-period comets with perihelia in the range $\sim 1-2$ au.

Sensitivity to exposed land on the early Earth

An important pre-requisite for the delivery of a prebiotically relevant concentration of HCN is impact onto exposed, subaerial land. Moreover, we note that many prebiotic chemical networks rely on short-term wet-dry cycling to drive the biogeochemical reactions required for the synthesis of key biopolymers[19,20]. However, despite its central importance for many origins of life scenarios, the extent of exposed land on the early Earth remains poorly constrained[21]. Further, while there exist many models of continental growth on the early Earth [see[22] and references therein], these are not direct proxies for exposed landmass, given the non-monotonic history of ocean volume[21].

In the interest of simplicity, we therefore adopt the McCulloch & Bennett[23] model of continental growth as a proxy for exposed landmass (following[19]). While this is likely a large overestimate of exposed landmass, given the potential for global oceans to limit subaerial landmass to hotspot oceanic islands[24], this guarantees that our results represent a best-case scenario for the cometary delivery of prebiotic feedstock molecules.

If we were instead to adopt the Guo & Korenaga[25] model of continental growth, which favours much quicker crustal formation compared to the rather than McCulloch & Bennett[23] model (Fig. 2), the number of impacts delivering > 10 mM HCN increases only to 1 (Table 1). This indicates that our results are insensitive to the significant uncertainty in models of continental growth, which is a consequence of the rapid decline in Earth's cometary impact flux[26], and the relative consensus between models regarding the very limited extent of exposed land during the early Hadean.

Sensitivity to dynamical model of Earth's cometary bombardment

Monte Carlo simulations using the Brasser et al.,[27] impact chronology. Despite our Monte Carlo simulations, when adopting the Nesvorný et al.[26] chronology, recording total cometary mass accretion of $(2.63 \pm 0.10) \times 10^{18}$ kg – consistent with independent constraints on Earth's cometary accretion[28,29] – we could equally have adopted an alternative model of Earth's cometary bombardment. This choice will, in principle, change the velocity distribution and timing of cometary impacts onto Earth, and therefore has the potential to affect the number of successful cometary impacts.

Here, we test our sensitivity to the assumed dynamical model of cometary bombardment, adopting instead the Brasser et al.[27] impact chronology. This study determines the initial conditions most likely to reproduce the modern-day architecture of the Solar System's giant planets, and given these initial conditions calculates the fraction of comets in the trans-Neptunian disk that enter the inner Solar System. The temporal mass of comets crossing 1.7 au is parametrised as

$$M_c = 4M_E \sum_{i=1}^{3} a_i \exp\left(\frac{4500 - t}{\tau_i}\right), \tag{14}$$

where a_i and τ_i are fitting constants, and M_E is the mass of the Earth. Cometary mass accretion to the early Earth is encapsulated via a mean impact probability, $p_{\text{imp}} = 3.1 \times 10^{-8}$. This corresponds to a total mass accretion of 1.1×10^{19} kg, again roughly consistent with available isotopic constraints[28,29].

At each timestep, t_i, in our Monte Carlo simulation, total cometary mass accretion to the Earth is therefore

$$\delta m_i = [M_c(t_i) - M_c(t_{i+1})]\, p_{\text{imp}}. \tag{15}$$

Given the steady-state SFD of Earth-crossing comets, this determines the constants of proportionality K_i at each timestep via

$$\delta m_i = \frac{\pi \rho}{6} \left\{ K_1 \int_{0.1}^{1} D^{3-\alpha_1} dD + K_2 \int_{1}^{50} D^{3-\alpha_2} dD + K_3 \int_{50}^{150} D^{3-\alpha_3} dD + K_4 \int_{150}^{1000} D^{3-\alpha_4} dD \right\}, \tag{16}$$

and the continuity of the SFD. The mean number of cometary impacts, $\bar{N}_i$ is then

$$\bar{N}_i = K_1 \int_{0.1}^{1} D^{-\alpha_1} dD + K_2 \int_{1}^{50} D^{-\alpha_2} dD + K_3 \int_{50}^{150} D^{-\alpha_3} dD + K_4 \int_{150}^{1000} D^{-\alpha_4} dD. \tag{17}$$

As before, this will not typically be an integer, and we therefore determine the number of cometary impacts, N_i, by sampling from a Poisson distribution with mean value $\bar{N}_i$.

The Brasser et al.[27] simulations record a much lower mean impact velocity of only $\langle v_{\text{imp}} \rangle = 22\ \text{km}\,\text{s}^{-1}$, which we use in our Monte Carlo simulations. A by-product of the much lower mean velocity is an increase in the collisional impact probability[30]; relative to the Nesvorný et al.[26] chronology, there is a rapid decrease in Earth's cometary impact flux. The results of this comparison are shown in Fig. 3, which clearly demonstrates the more rapid decline impact flux in the Brasser et al.[27] chronology.

Despite the greater overall cometary mass accretion, and lower mean impact velocity, the median number of cometary impact delivering > 10 mM HCN is still 0 – exactly the same as with the Nesvorný et al.[26] chronology. The distribution in total number of successful impacts is shown in Fig. 4, highlighting that the number of simulations recording no successful cometary impacts is in fact substantially greater with the Brasser et al.[27] chronology. This is a consequence of the rapid decline in impact flux, which exacerbates the tension with the slow growth of exposed landmass during the early Hadean[21]. The results of this comparison are thus reassuring, with two independent dynamical models of Earth's cometary bombardment in agreement that there was, in this best-case scenario, one successful impact at very early times.

Sensitivity to assumed mechanism of comet fragmentation

Motivated by the results of dynamical simulations of short-period comet formation[31,32], and the short physical lifetime of JFCs[33], we numerically modelled the effects of cometary fragmentation on the SFD of Earth-crossing comets. While recent work indicates that rotational instability is a leading mechanism responsible for the the disruption of short-period comets[10], it is likely that other mechanisms contribute to the disruption of short-period comets [see[34] for a review of comet fragmentation].

Previous work[35] has remained agnostic to the responsible physical mechanism, adopting instead the Di Sisto et al.[31] prescription of comet fragmentation. This model assumes a constant probability of fragmentation per perihelion passage, and instead takes into account the fact that it will be harder for fragments to gravitationally escape from large comets (given that escape velocity $v_{\rm esc} = \sqrt{2GM/R} \propto R$ is proportional to radius). Small comets, with minimal self-gravity, are unlikely to retain significant mass following fragmentation. The slope of the steady-state SFD remains essentially unchanged if we were to instead use the Di Sisto et al.[31] model of comet fragmentation. The results of our Monte Carlo calculation remain unchanged when using the Di Sisto et al.[31] model of cometary fragmentation; with, on average, 0 cometary impacts delivering > 10 mM HCN.

Sensitivity to atmospheric surface density

Given uncertainty in atmospheric surface density during the Hadean and Archean epochs, we report here the results of sensitivity tests in which we vary atmospheric surface density. While constraints on the atmospheric evolution of the Hadean Earth are scarce, and remain heavily debated, we note that numerical modelling suggests that it was initially very dense (~ 100 bar) following the degassing of Earth's magma ocean[36]). It is thought that there was then a quick transition to a surface pressure of $\lesssim 1.1$ bar during the Archean.

To encompass plausible variation in atmospheric surface density, we therefore re-ran our Monte Carlo simulations with surface density in the range $\rho_{\rm atm} \in [0.1, 10]\ \rho_{\rm atm,0}$, where $\rho_{\rm atm,0} = 1.225\ {\rm kg\,m^{-3}}$ is the canonical value we consider in the main text. The results of our Monte Carlo calculation are summarised in Table 2, in which it can be seen that the number of successful impacts are consistent within error, suggesting that our key conclusions are robust to this model assumption.

We highlight, however, that it is possible that our results for the $\rho_{\rm atm} = 0.1\ \rho_{\rm atm,0}$ simulation potentially underestimate the true number of successful impacts, given that it is likely that some $D < 100$ m comets would survive atmospheric entry[37].Since the McDonald et al.,[38] parameterisation does not extend to this size range, it was not possible to determine HCN delivery from this subset of comets. Nonetheless, given that the comet size-frequency distribution is very shallow in this size range (see figure 1, main text), we do not expect this to apply to a significant population of comets.

Which comets deliver prebiotically relevant concentrations of HCN?

To highlight the subset of comets incident at the top of Earth's (and indeed also Mars and Venus) atmosphere responsible for the delivery of a prebiotically relevant concentration of HCN, we compare in Figs. 5–7 the distribution of comets at the top-of-atmosphere with the population that subsequently deliver > 10 mM HCN.

For Earth and Mars, it is evident that the impact velocity distribution of comets incident on each planet largely controls the survival of HCN during hypervelocity impact. To moderate the peak temperatures – and associated destruction of HCN – highly-oblique impacts are required. This is seen clearly in Figs. 5 and 6 for which there are tight correlations between impact velocity and angle for those comets delivering a prebiotically relevant concentration of HCN. The dominant role played by entry angle is evidenced by the distribution of entry angles of successful cometary impacts, which bears little resemblance to the overall distribution of comets arriving at the top-of-atmosphere.

The picture is more complicated for Venus, which has significantly less cometary impacts delivering a prebiotically relevant concentration of HCN relative to both Mars and Earth. The key differences are driven by Venus' dense atmosphere, which drives the destruction of very large km-scale comets. Perhaps counter-intuitively, the velocity distribution of comets appears to make little effect on the survival of HCN. It is instead the size of comets controlling the delivery of high concentrations of HCN; comets with initial radius $\sim$ 3 km, aero-braked to very low velocities, that dominate the delivery of prebiotically relevant concentrations of HCN. This corresponds to a vanishingly small corner of parameter space, sampling the largest sizes in the comets' SFD, and it remains a possibility that there is additionally the significant destruction of HCN during the fragmentation of these comets near to Venus' surface[37].

Sensitivity to required HCN concentration

For comparison with recent experimental studies, we quantify successful cometary impacts by the maximum concentration of HCN delivered to local, subaerial environments. In Figs. 3 and 4 (of the main text) we require the delivery of $\gtrsim 10$ mM HCN for successful subsequent prebiotic chemistry, motivated by (i) the synthesis of adenine with 11.1 mM HCN[39], and (ii) the synthesis of simple sugars and amino acid precursors from 200 mM KCN in the cyanosulfidic scenario[40]. Indeed, in support of this choice, many recent laboratory studies use $10 - 100$ mM HCN[41–43]. As discussed in the main text, this is a conservative choice and represents a best-case for cometary delivery, assuming the dilution of surviving HCN in only the comet's water

ice: several environmental processes will act to rapidly dilute cometary HCN below productive concentrations for prebiotic chemistry.

Despite representing a conservative choice, here we demonstrate our conclusions are insensitive to this choice of critical concentration. Considering three concentrations, 0.1 mM, 1 mM, and 10 mM, the median number of successful cometary impacts are 16, 5, and 0 respectively (see also Fig. 8 for comparison with Fig. 3 of the main text). Despite the two orders of magnitude decrease in HCN concentration, the number of successful cometary impacts increases only modestly. Assuming, optimistically, there were 50 successful cometary impacts, the heuristic origins of life timescale (see Eq. 2) changes insignificantly. Assuming the end-member values of $p_{\rm abio} \simeq 10^3$ Gyr^{-1} and $\tau_{\rm HCN} = 1$ kyr, we find $\tau_{\rm ool} \simeq 10$ Gyr – far in excess of the age of the Solar System.

To summarise, while there clearly remains uncertainty in the concentration of HCN required for successful prebiotic chemistry – which may require detailed aqueous kinetics models grounded in geochemistry to be accurately constrained[44] – our conclusions are reassuringly insensitive to this uncertainty. Indeed, given the rapid dilution of cometary HCN in subaerial environments, we likely overestimate the true concentration of HCN post cometary impact, which we expect to be more significant following the delivery of $\lesssim 10$ mM HCN.

Maximum HCN concentration following cometary impact

Here we describe the calculation of the maximum potential concentration of HCN delivered by cometary impact, which we quote as ~ 30 mM in the main text. To calculate the maximum possible HCN concentration, and present the best-case scenario for the cometary delivery of HCN, we assume the dilution of HCN in only the comet's water ice. The number of moles of water per litre delivered by the comet, is given by

$$n_{\rm H_2O} = \frac{f_w \rho_{\rm com}}{M_{\rm H_2O}}, \tag{18}$$

where f_w is the comets' water-ice mass fraction, $\rho_{\rm com}$ its bulk density and $M_{\rm H_2O}$ the molar mass of water. Given a HCN abundance $c_{\rm HCN} = 10^{-3}$ (following[45]) the maximum HCN concentration is thus

$$[\mathrm{HCN}] = c_{\rm HCN} n_{\rm H_2O} = 33\ f_w \left(\frac{\rho_{\rm com}}{0.6\ \mathrm{g\,cm^{-3}}}\right)\ \mathrm{mM} \tag{19}$$

where we assume a bulk density of $\rho_{\rm com} = 0.6\ \mathrm{g\,cm^{-3}}$ (following[1]).

Given uncertainty in the water-ice mass fraction of comets[46], and to ensure that our results provide a best-case scenario for cometary delivery (in-keeping with other model assumptions made throughout) we assume $f_w = 1$, which corresponds to an HCN concentration of approximately 30 mM. In practice, the non-zero refractory-to-ice mass ratio[46], and environmental dilution of cometary HCN (see Discussion) combine such that the cometary delivery of a prebiotically relevant concentration of HCN was an exceptionally rare event.

Exoplanet escape velocities

To demonstrate the radius-dependence of planetary escape velocity, we use data from the PlanetS catalogue (`https://dace.unige.ch/`), which comprises 860 exoplanets with well-characterised masses and radii (i.e., relative measurement uncertainties less than 25% and 8% respectively). The distribution of this catalogue of exoplanets in mass-radius space is shown in Fig. 9, with the (piece-wise) data-driven mass-radius relationship from Müller et al.[47] included for reference. For Earth-like exoplanets (i.e., masses less than roughly $4M_E$), the escape velocity scales as

$$v_{\rm esc} = \sqrt{\frac{2GM_{\rm pl}}{R_{\rm pl}}} \propto M_{\rm pl}^{0.365} \tag{20}$$

an increasing function of exoplanet mass. Escape velocity as a function of planet radius is shown in the lower panel of Fig. 9; only 5 exoplanets in the PlanetS catalogue (0.58%) have escape velocity less than Earth's. These are all above Mars' escape velocity, with the lowest escape velocity in the sample 7.4 $\mathrm{km\,s^{-1}}$.

References

1. Kokotanekova, R. *et al.* Rotation of cometary nuclei: new light curves and an update of the ensemble properties of Jupiter-family comets. *Mon. Notices Royal Astron. Soc.* **471**, 2974–3007, DOI: 10.1093/mnras/stx1716 (2017).
2. Safrit, T. K. *et al.* The Formation of Bilobate Comet Shapes through Sublimative Torques. *Planet Sci. J.* **2**, 14, DOI: 10.3847/PSJ/abc9c8 (2021).

3. Davidsson, B. J. R. Tidal Splitting and Rotational Breakup of Solid Spheres. *Icarus* **142**, 525–535, DOI: 10.1006/icar.1999.6214 (1999).
4. Davidsson, B. J. R. Tidal Splitting and Rotational Breakup of Solid Biaxial Ellipsoids. *Icarus* **149**, 375–383, DOI: 10.1006/icar.2000.6540 (2001).
5. Sekanina, Z. Formation and Evolution of the Stream of SOHO Kreutz Sungrazers. II. Results and Implications of Monte Carlo Simulation. *arXiv e-prints* arXiv:2404.00887, DOI: 10.48550/arXiv.2404.00887 (2024).
6. Warner, B. D., Harris, A. W. & Pravec, P. The asteroid lightcurve database. *Icarus* **202**, 134–146, DOI: 10.1016/j.icarus.2009.02.003 (2009).
7. Attree, N. *et al.* Tensile strength of 67P/Churyumov-Gerasimenko nucleus material from overhangs. *Astron. & Astrophys.* **611**, A33, DOI: 10.1051/0004-6361/201732155 (2018).
8. Groussin, O. *et al.* The Thermal, Mechanical, Structural, and Dielectric Properties of Cometary Nuclei After Rosetta. *Space Sci. Rev.* **215**, 29, DOI: 10.1007/s11214-019-0594-x (2019).
9. Binzel, R. P., Xu, S., Bus, S. J. & Bowell, E. Origins for the Near-Earth Asteroids. *Science* **257**, 779–782, DOI: 10.1126/science.257.5071.779 (1992).
10. Jewitt, D. Systematics and Consequences of Comet Nucleus Outgassing Torques. *The Astron. J.* **161**, 261, DOI: 10.3847/1538-3881/abf09c (2021).
11. Whipple, F. L. A comet model. I. The acceleration of Comet Encke. *The Astrophys. J.* **111**, 375–394, DOI: 10.1086/145272 (1950).
12. Marsden, B. G., Sekanina, Z. & Yeomans, D. K. Comets and nongravitational forces. V. *The Astron. J.* **78**, 211, DOI: 10.1086/111402 (1973).
13. Jewitt, D. Cometary Rotation: an Overview. *Earth Moon Planets* **79**, 35–53, DOI: 10.1023/A:1006272914117 (1997).
14. Samarasinha, N. H. & Mueller, B. E. A. Relating Changes in Cometary Rotation to Activity: Current Status and Applications to Comet C/2012 S1 (ISON). *The Astrophys. J. Lett.* **775**, L10, DOI: 10.1088/2041-8205/775/1/L10 (2013).
15. Rafikov, R. R. Non-Gravitational Forces and Spin Evolution of Comets. *arXiv e-prints* arXiv:1809.05133, DOI: 10.48550/arXiv.1809.05133 (2018).
16. Kokotanekova, R. *et al.* Implications of the small spin changes measured for large Jupiter-family comet nuclei. *Mon. Notices Royal Astron. Soc.* **479**, 4665–4680, DOI: 10.1093/mnras/sty1529 (2018).
17. Jewitt, D. Destruction of Long-period Comets. *The Astron. J.* **164**, 158, DOI: 10.3847/1538-3881/ac886d (2022).
18. Sosa, A. & Fernández, J. A. Cometary masses derived from non-gravitational forces. *Mon. Notices Royal Astron. Soc.* **393**, 192–214, DOI: 10.1111/j.1365-2966.2008.14183.x (2009).
19. Pearce, B. K. D., Pudritz, R. E., Semenov, D. A. & Henning, T. K. Origin of the RNA world: The fate of nucleobases in warm little ponds. *Proc. Natl. Acad. Sci.* **114**, 11327–11332, DOI: 10.1073/pnas.1710339114 (2017).
20. Campbell, T. D. *et al.* Prebiotic condensation through wet–dry cycling regulated by deliquescence. *Nat. Commun.* **10**, 4508 (2019).
21. Korenaga, J. Was There Land on the Early Earth? *Life* **11**, 1142, DOI: 10.3390/life11111142 (2021).
22. Korenaga, J. Hadean geodynamics and the nature of early continental crust. *Precambrian Res.* **359**, 106178, DOI: 10.1016/j.precamres.2021.106178 (2021).
23. McCulloch, M. T. & Bennett, V. C. Evolution of the early Earth: Constraints from ^{143}Nd ^{142}Nd isotopic systematics. *Lithos* **30**, 237–255, DOI: 10.1016/0024-4937(93)90038-E (1993).
24. Bada, J. L. & Korenaga, J. Exposed Areas Above Sea Level on Earth >3.5 Gyr Ago: Implications for Prebiotic and Primitive Biotic Chemistry. *Life* **8**, 55, DOI: 10.3390/life8040055 (2018).
25. Guo, M. & Korenaga, J. Argon constraints on the early growth of felsic continental crust. *Sci. Adv.* **6**, eaaz6234, DOI: 10.1126/sciadv.aaz6234 (2020).
26. Nesvorný, D. *et al.* Early bombardment of the moon: Connecting the lunar crater record to the terrestrial planet formation. *Icarus* **399**, 115545, DOI: 10.1016/j.icarus.2023.115545 (2023).
27. Brasser, R., Werner, S. C. & Mojzsis, S. J. Impact bombardment chronology of the terrestrial planets from 4.5 Ga to 3.5 Ga. *Icarus* **338**, 113514, DOI: 10.1016/j.icarus.2019.113514 (2020).

28. Marty, B. *et al.* Xenon isotopes in 67P/Churyumov-Gerasimenko show that comets contributed to Earth's atmosphere. *Science* **356**, 1069–1072, DOI: 10.1126/science.aal3496 (2017).

29. Cassata, W. S. A refined isotopic composition of cometary xenon and implications for the accretion of comets and carbonaceous chondrites on earth. *Earth Planet. Sci. Lett.* **660**, 119307, DOI: https://doi.org/10.1016/j.epsl.2025.119307 (2025).

30. Wetherill, G. W. Collisions in the Asteroid Belt. *J. Geophys. Res.* **72**, 2429, DOI: 10.1029/JZ072i009p02429 (1967).

31. Di Sisto, R. P., Fernández, J. A. & Brunini, A. On the population, physical decay and orbital distribution of Jupiter family comets: Numerical simulations. *Icarus* **203**, 140–154, DOI: 10.1016/j.icarus.2009.05.002 (2009).

32. Nesvorný, D. *et al.* Origin and Evolution of Short-period Comets. *The Astrophys. J.* **845**, 27, DOI: 10.3847/1538-4357/aa7cf6 (2017).

33. Levison, H. F. & Duncan, M. J. From the Kuiper Belt to Jupiter-Family Comets: The Spatial Distribution of Ecliptic Comets. *Icarus* **127**, 13–32, DOI: 10.1006/icar.1996.5637 (1997).

34. Boehnhardt, H. & Binzel, R. P. *Split Comets*, 301–316 (University of Arizona Press, 2004).

35. Rigley, J. K. & Wyatt, M. C. Comet fragmentation as a source of the zodiacal cloud. *Mon. Notices Royal Astron. Soc.* **510**, 834–857, DOI: 10.1093/mnras/stab3482 (2022).

36. Elkins-Tanton, L. T. Linked magma ocean solidification and atmospheric growth for Earth and Mars. *Earth Planet. Sci. Lett.* **271**, 181–191, DOI: 10.1016/j.epsl.2008.03.062 (2008).

37. Anslow, R. J. *et al.* The atmospheric entry of cometary impactors. *Mon. Notices Royal Astron. Soc.* **539**, 376–392, DOI: 10.1093/mnras/staf507 (2025).

38. McDonald, C. H. *et al.* Constraining the survival of hcn during cometary impacts. *Icarus* 116704, DOI: https://doi.org/10.1016/j.icarus.2025.116704 (2025).

39. Oró, J. & Kimball, A. Synthesis of purines under possible primitive earth conditions. i. adenine from hydrogen cyanide. *Arch. Biochem. Biophys.* **94**, 217–227, DOI: https://doi.org/10.1016/0003-9861(61)90033-9 (1961).

40. Xu, J. *et al.* Photochemical reductive homologation of hydrogen cyanide using sulfite and ferrocyanide. *Chem. Commun.* **54**, 5566–5569, DOI: 10.1039/C8CC01499J (2018).

41. Ritson, D. & Sutherland, J. D. Prebiotic synthesis of simple sugars by photoredox systems chemistry. *Nat. Chem.* **4**, 895–899 (2012).

42. Ritson, D. J. & Sutherland, J. D. Thiophosphate photochemistry enables prebiotic access to sugars and terpenoid precursors. *Nat. Chem.* **15**, 1470–1477 (2023).

43. Yi, R., Hongo, Y., Yoda, I., Adam, Z. R. & Fahrenbach, A. C. Radiolytic synthesis of cyanogen chloride, cyanamide and simple sugar precursors. *ChemistrySelect* **3**, 10169–10174, DOI: https://doi.org/10.1002/slct.201802242 (2018). https://chemistry-europe.onlinelibrary.wiley.com/doi/pdf/10.1002/slct.201802242.

44. White, S. B. & Rimmer, P. B. Do-Nothing prebiotic chemistry: Chemical kinetics as a window into prebiotic plausibility. *Acc. Chem. Res.* **58**, 1–10 (2025).

45. Mumma, M. J. & Charnley, S. B. The Chemical Composition of Comets—Emerging Taxonomies and Natal Heritage. *Annu. Rev. Astron. Astrophys.* **49**, 471–524, DOI: 10.1146/annurev-astro-081309-130811 (2011).

46. Choukroun, M. *et al.* Dust-to-Gas and Refractory-to-Ice Mass Ratios of Comet 67P/Churyumov-Gerasimenko from Rosetta Observations. *Space Sci. Rev.* **216**, 44, DOI: 10.1007/s11214-020-00662-1 (2020).

47. Müller, S., Baron, J., Helled, R., Bouchy, F. & Parc, L. The mass-radius relation of exoplanets revisited. *Astron. & Astrophys.* **686**, A296, DOI: 10.1051/0004-6361/202348690 (2024). 2311.12593.

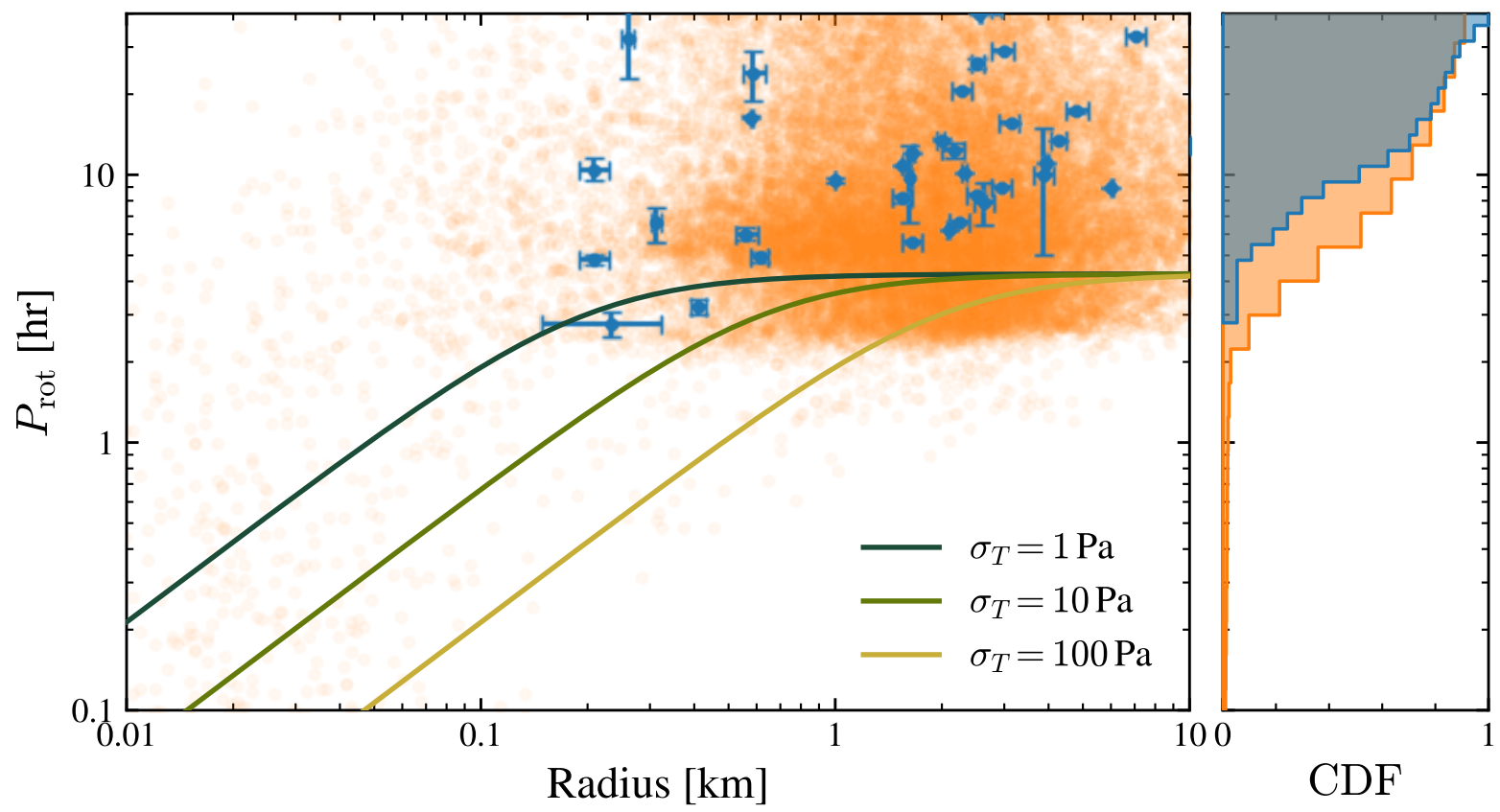

Figure 1. Modern Jupiter-family comets' radius-rotation period distribution is strongly sculpted by rotational instability. Cometary rotation periods and radii are taken from[1] (blue), while the rotation periods and radii of small asteroids are from[6] (orange); the cumulative distribution function (CDF) of rotation periods is shown in the right-hand panel. The minimum rotation periods stable to rotational instability are included for three representative tensile strengths in the range $1-100$ Pa; all comets in this sample are stable to rotational instability. The small asteroids distribution shows significant differences to JFCs, consistent with a significantly increased tensile strength of small asteroids.

Land fraction model	$c_{\rm HCN}$ [mM]	Median N	$P(N=0)$	$P(N=1)$	$P(N>1)$	Timing of last successful impact [Gya]
McCulloch & Bennett (1993)	0.1	16^{+4}_{-4}	0.00	0.00	1.00	$4.54^{+0.01}_{-0.02}$
	1.0	5^{+3}_{-2}	0.00	0.02	0.97	$4.52^{+0.02}_{-0.07}$
	10	0^{+1}_{-0}	0.55	0.33	0.13	$4.41^{+0.11}_{-0.29}$
	30	0^{+0}_{-0}	1.00	0.00	0.00	$4.25^{+0.25}_{-0.43}$
Guo & Korenaga (2020)	0.1	32^{+6}_{-7}	0.00	0.00	1.00	$4.19^{+0.02}_{-0.03}$
	1.0	13^{+4}_{-4}	0.00	0.00	1.00	$4.17^{+0.03}_{-0.04}$
	10	1^{+2}_{-1}	0.32	0.31	0.37	$4.07^{+0.09}_{-0.20}$
	30	0^{+0}_{-0}	0.99	0.01	0.00	$3.94^{+0.16}_{-0.24}$

Table 1. Summary statistics for the delivery of $> c_{\rm HCN}$ mM HCN to Earth when varying the assumed model of continental growth. We use the near end-member cases of the McCulloch & Bennet[23] and Guo & Korenaga[25] models as proxies for the slow and rapid growth of continental crust respectively. We report the median number of successful impacts N (with 68 % confidence intervals), the probability of $N=0$, $N=1$, and $N>1$ successful impacts, and the most probable timing of successful cometary impact.

$\rho_{\rm atm}$ [1.225 kg m^{-3}]	Median number of successful impacts
0.1	0^{+1}_{-0}
1.0	0^{+1}_{-0}
10	3^{+1}_{-2}

Table 2. The median number of successful cometary impacts (delivering >10 mM HCN) as a function of atmospheric surface density, $\rho_{\rm atm}$. The errors on the number of successful cometary impacts report the 68 % confidence interval.

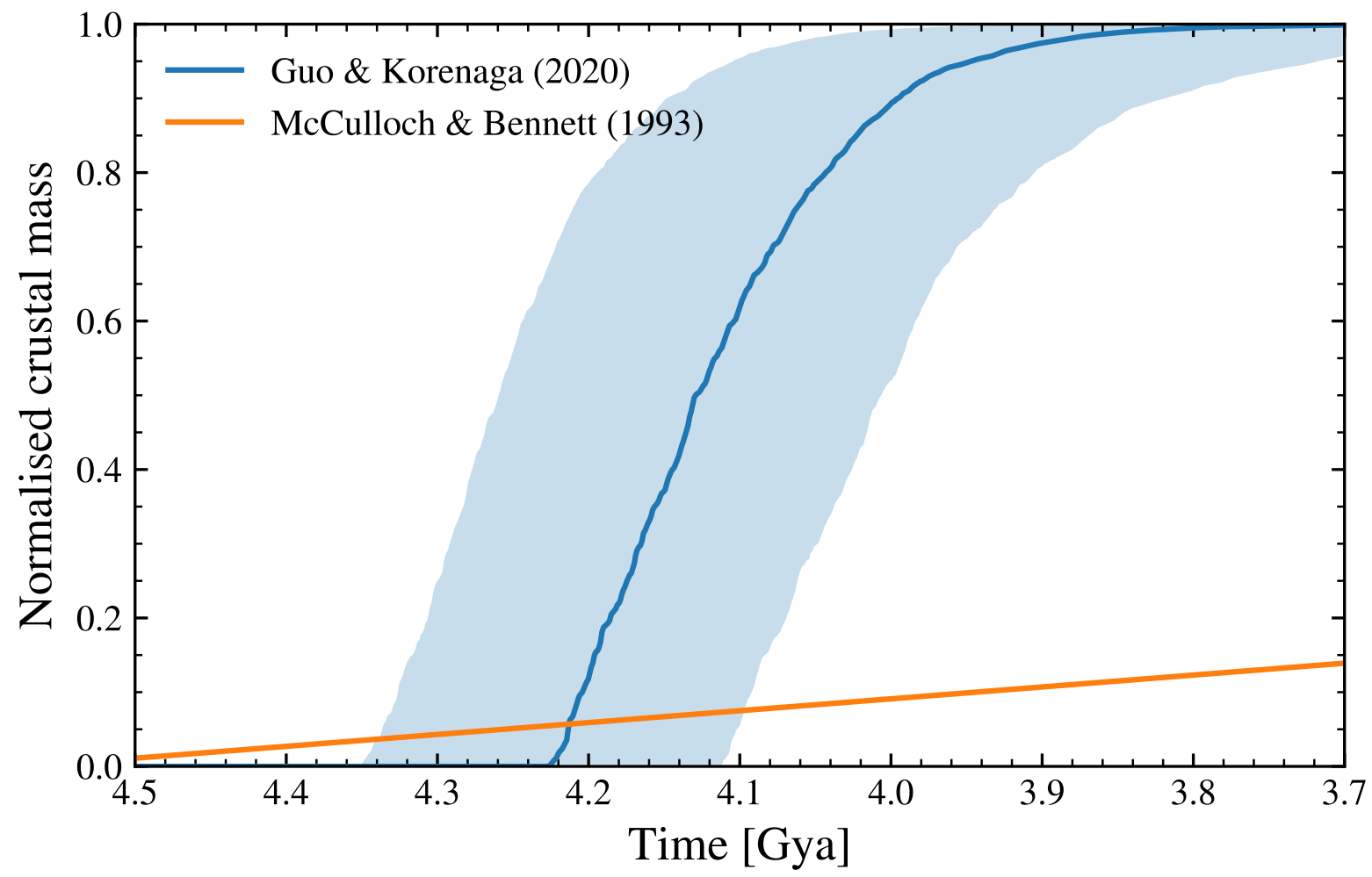

Figure 2. Models of continental growth on the Hadean Earth. Shown here are the McCulloch & Bennett[23] (orange) and Guo & Korenaga[25] (blue) models of continental growth, normalised to modern day continental crustal mass. These models are near end-member cases, describing respectively the slow and rapid growth of continental crust – an upper limit for subaerial land on the Hadean Earth. Only the central 50% of the Guo & Korenaga solutions are plotted for clarity.

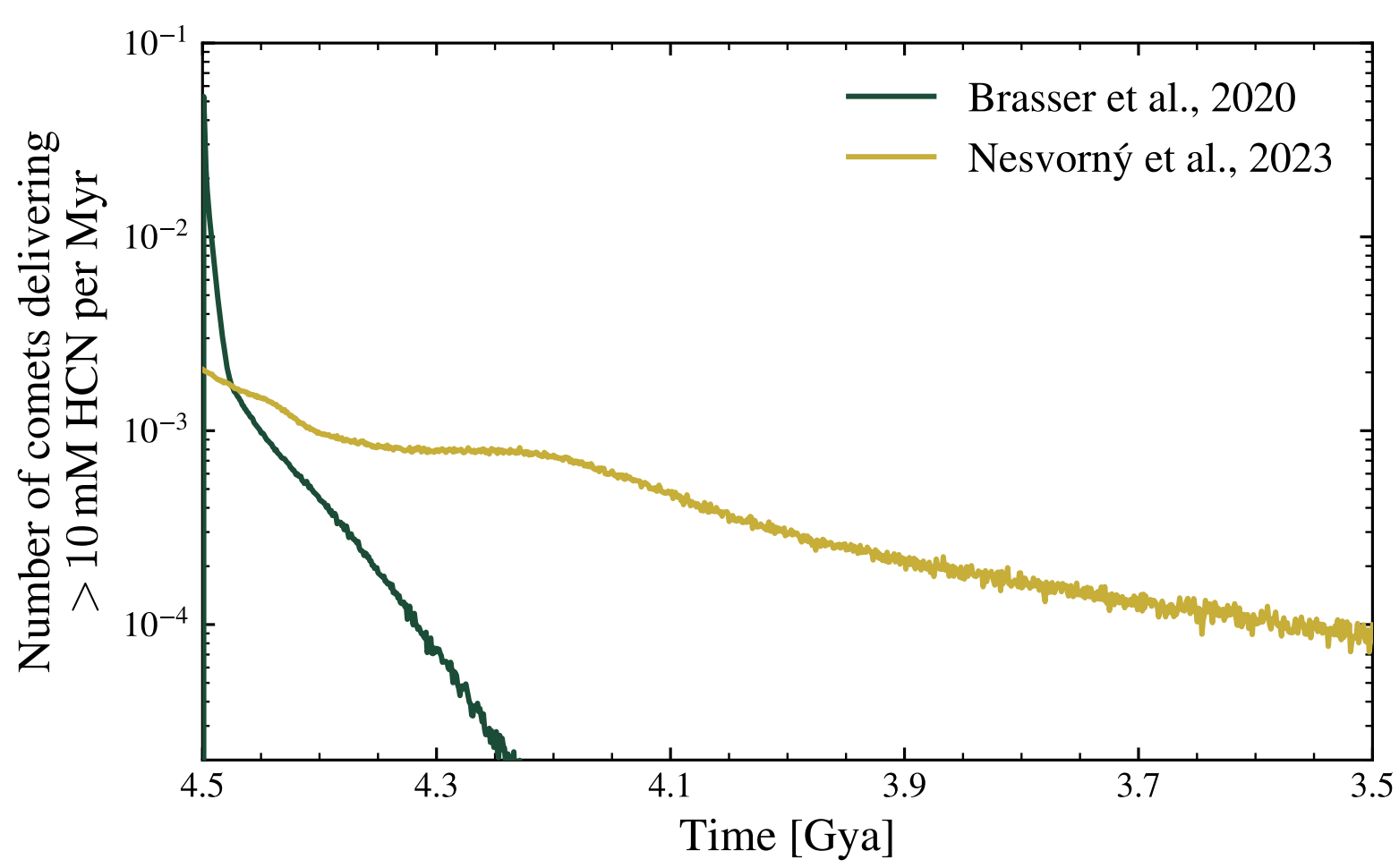

Figure 3. Results from Monte Carlo simulation of Earth's early cometary bombardment demonstrate little sensitivity to the assumed model of cometary bombardment. We vary only the dynamical model of Earth's cometary bombardment – using the Brasser et al.[27] chronology (blue) and Nesvorný et al.[26] chronology – and the comets' impact velocity distribution, keeping the assumed model of continental growth[23] constant. The median number of successful cometary impacts per Monte Carlo realisation is 0 in both cases, highlighting the insensitivity of our results to the assumed model of cometary bombardment.

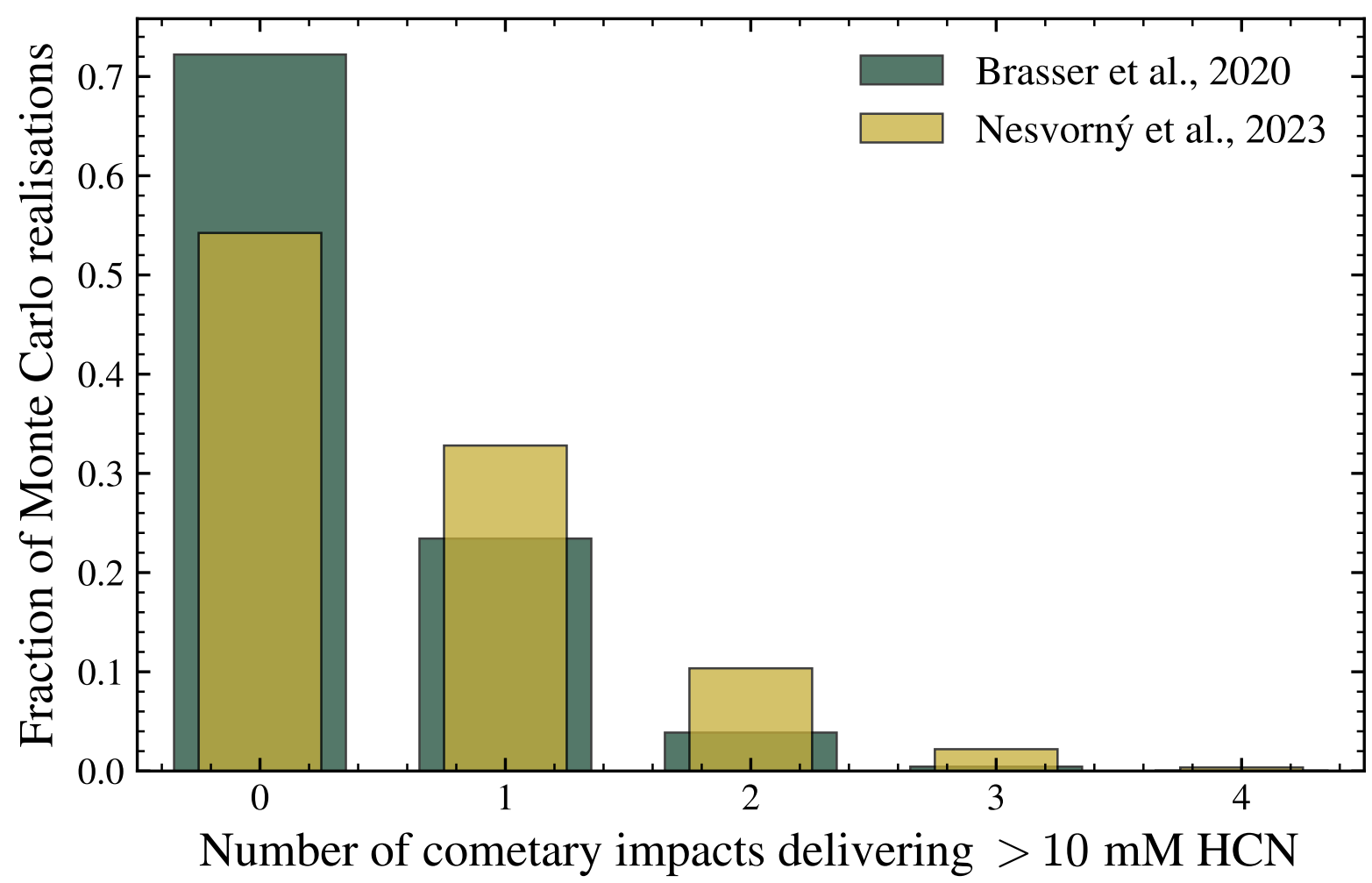

Figure 4. The distribution of successful cometary impacts per Monte Carlo simulation. The number of cometary impacts delivering > 10 mM HCN per Monte Carlo realisation of Earth's cometary bombardment history. The median number of successful impacts is zero, with 54 % (72 %) of simulations recording no successful cometary impacts for the Nesvorný (Brasser) impact chronology.

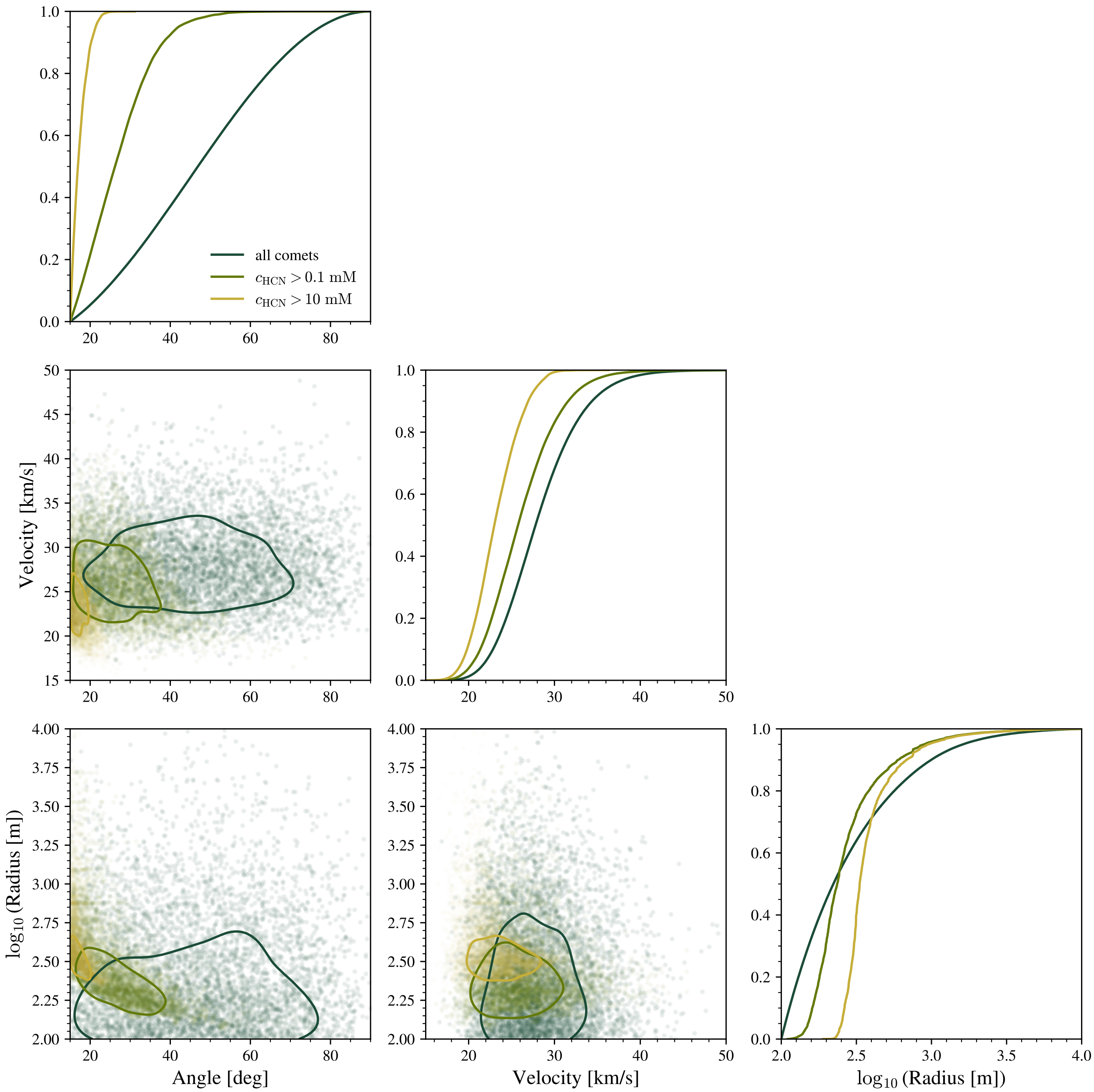

Figure 5. The subset of comets delivering prebiotically relevant concentrations of HCN to the Earth. The distribution of comets' impact velocities, sizes and entry angles at the top of the atmosphere (blue) is compared with the subset of these comets delivering 0.1 mM (green) and a prebiotically relevant concentration of HCN, 10 mM (yellow). The relationships between impact velocity, entry angle and initial radius are shown in the respective scatter plots.

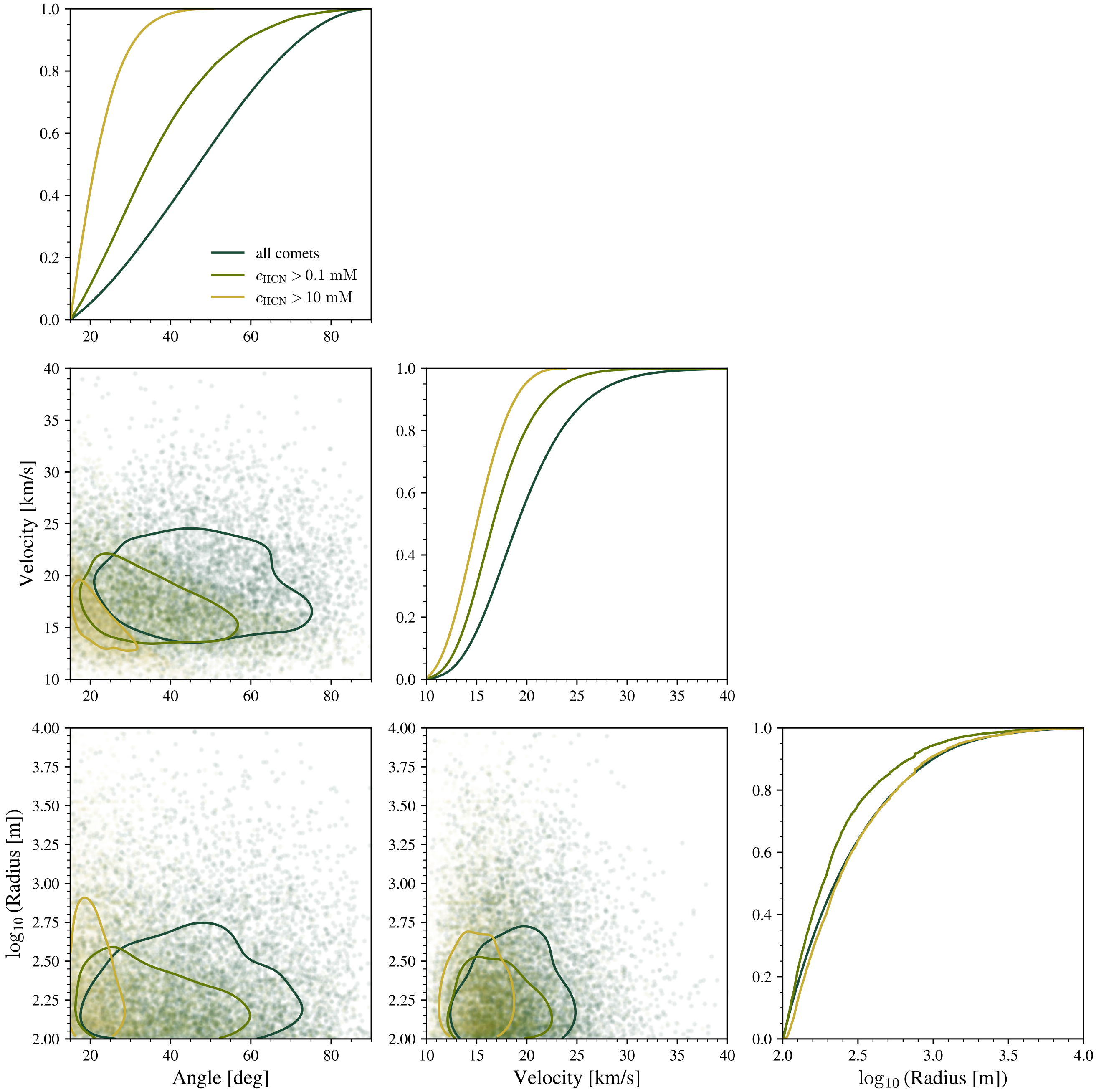

Figure 6. The subset of comets delivering prebiotically relevant concentrations of HCN to Mars. Same as Fig. 5, only for Mars – using Mars' cometary impact chronology, impact velocity distribution, and Mars' present-day 6 mbar atmospheric surface pressure.

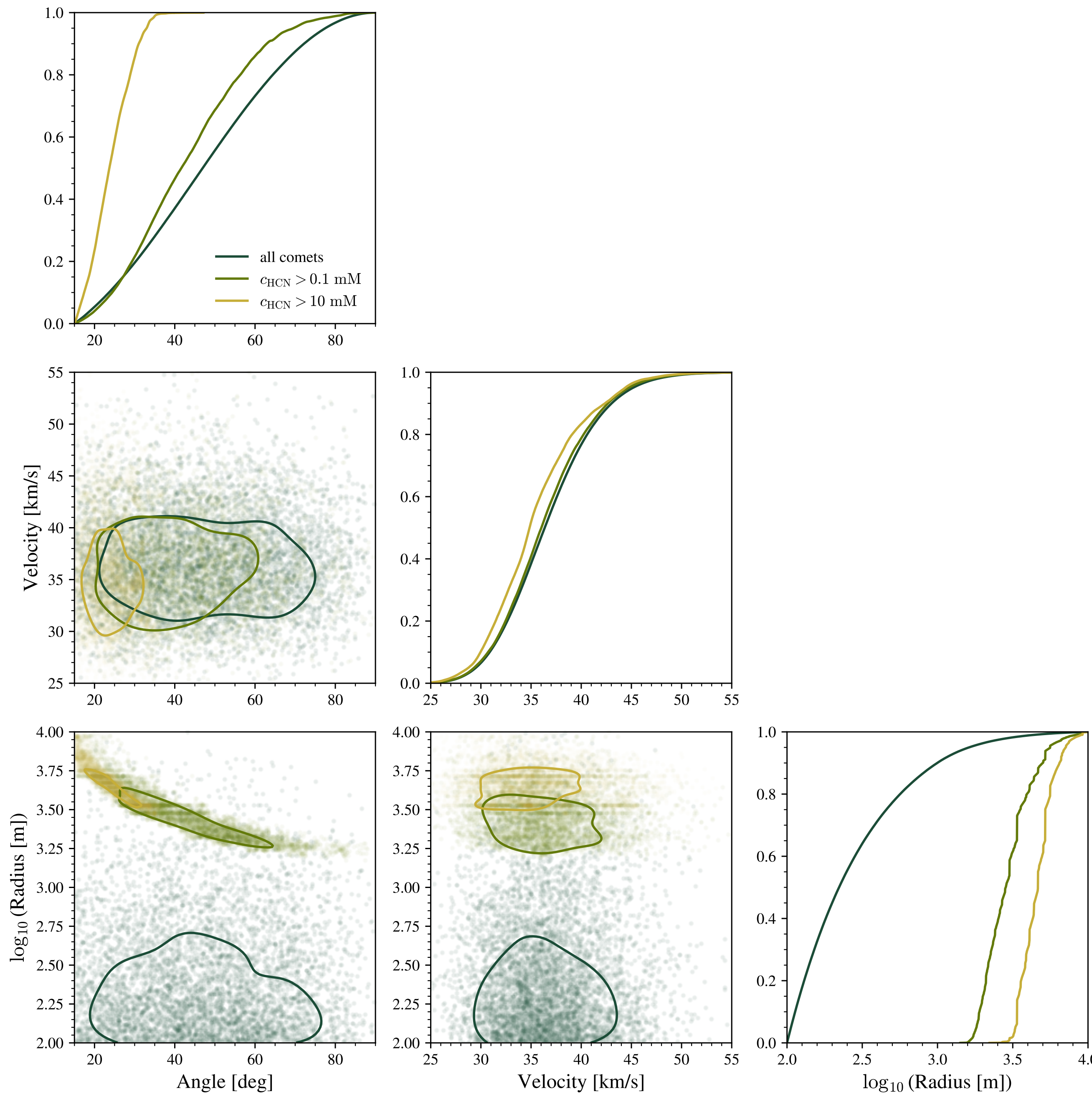

Figure 7. The subset of comets delivering prebiotically relevant concentrations of HCN to Venus. Same as Fig. 5, only for Venus – using Venus' cometary impact chronology, impact velocity distribution, and Venus' present-day 93 bar atmospheric surface pressure.

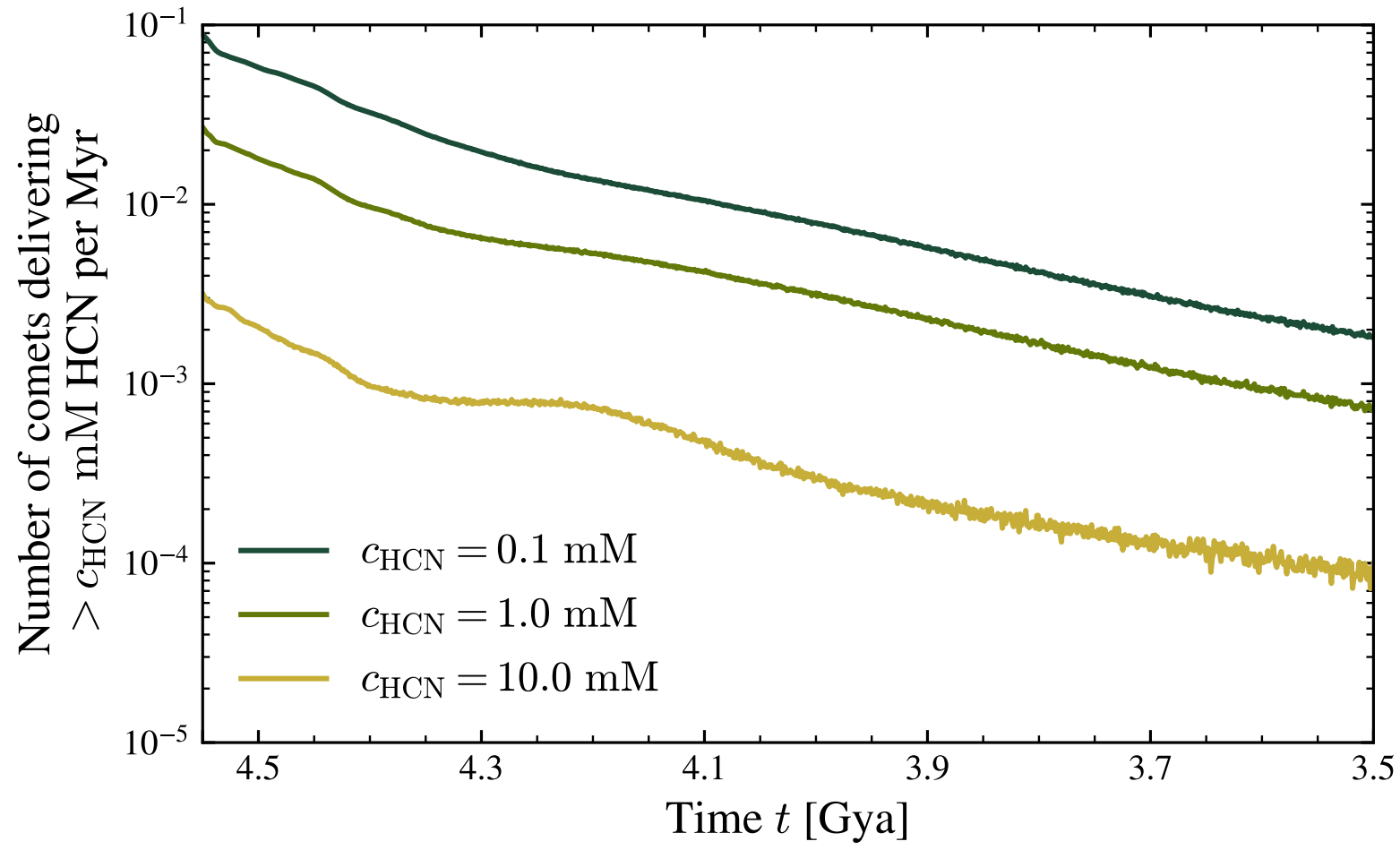

Figure 8. Results from Monte Carlo simulation of Earth's early cometary bombardment. The critical concentration of HCN, c_{HCN}, required for successful prebiotic chemistry is varied, considering $c_{HCN} = 0.1, 1.0, 10$ mM. The median number of successful cometary impacts per Monte Carlo realisation is 16, 5, and 0 respectively, increasing only modestly. Our conclusions are thus insensitive to the exact choice of c_{HCN}, and we argue present a best-case scenario for cometary delivery (see text).

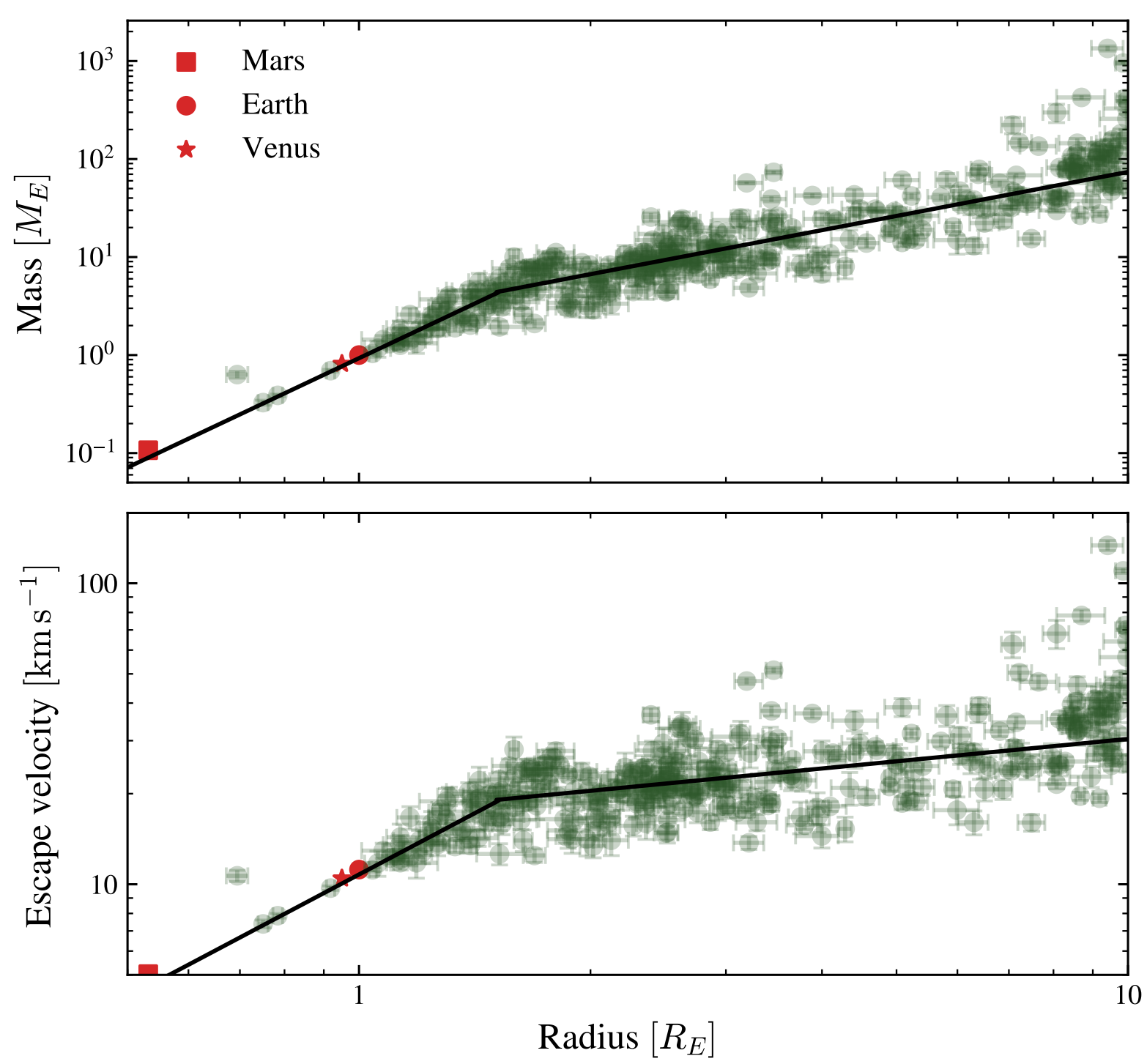

Figure 9. The mass-radius distribution (top) and escape-velocity-radius distribution (bottom) of the PlanetS exoplanet catalogue. Data from the PlanetS catalogue are displayed in green, with the Solar System's terrestrial planets included in red, and empirical mass-radius/escape velocity-radius relationships[47] included in black.
